# Supplementary material for: Exploratory Analysis of Molecular Subtypes in Early-Stage Osteosarcoma: Identifying Resistance and Optimizing Therapy
Source: Cancers (Basel). 2025 May 16;17(10):1677. doi: 10.3390/cancers17101677 (PMC12109990; doi:10.3390/cancers17101677)
Supplement: Supplementary file 1 [file cancers-17-01677-s001.zip › cancers-3551809-supplementary.pdf]

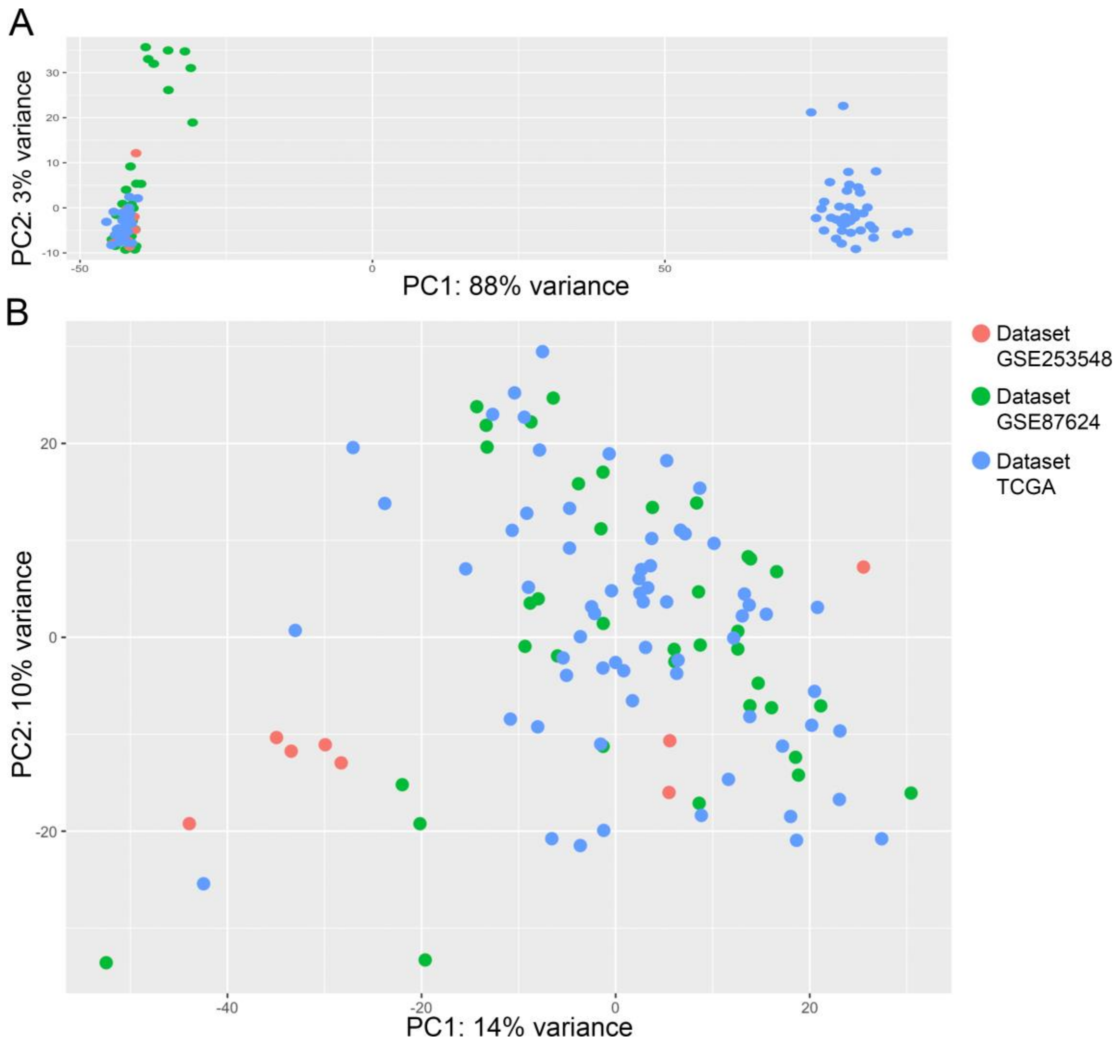

Figure S1. Principal component analysis (PCA) for the three datasets **A)** before and **B)** after batch normalisation.

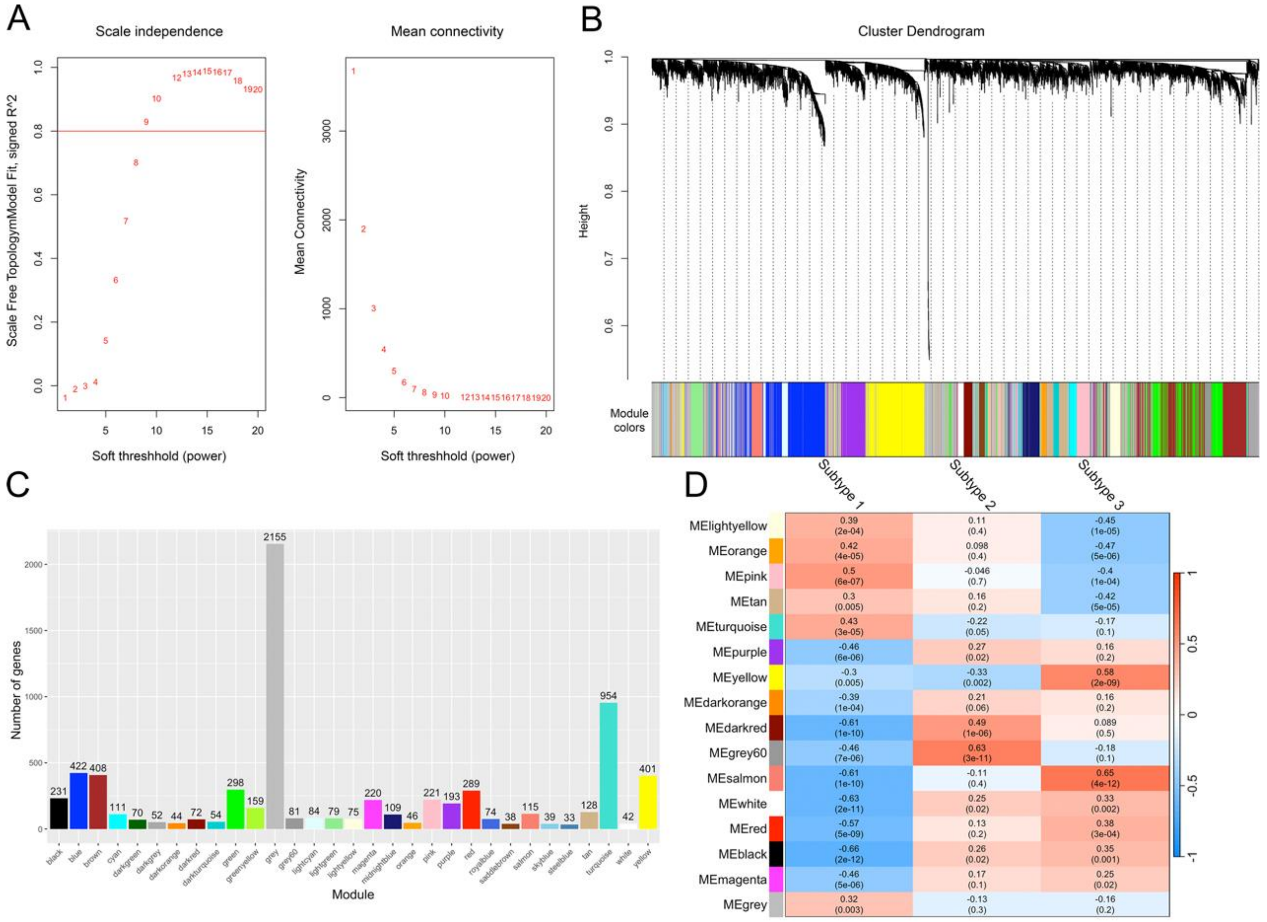

Figure S2. Construction and identification of modules associated with subtypes. **A)** Soft threshold selection based on scale independence and mean connectivity. **B)** Cluster dendrogram of 31 modules **C)** Number of genes in each gene co-expression module. **D)** Heatmap depicting correlations and adjusted P values between subtypes and identified modules.

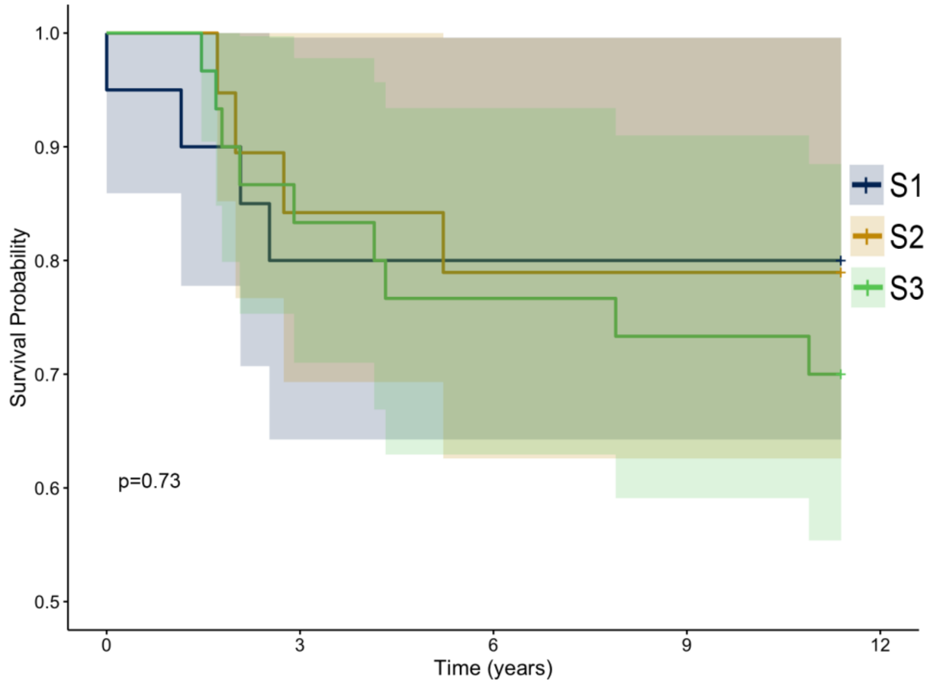

Figure S3. Survival probability of identified subtypes

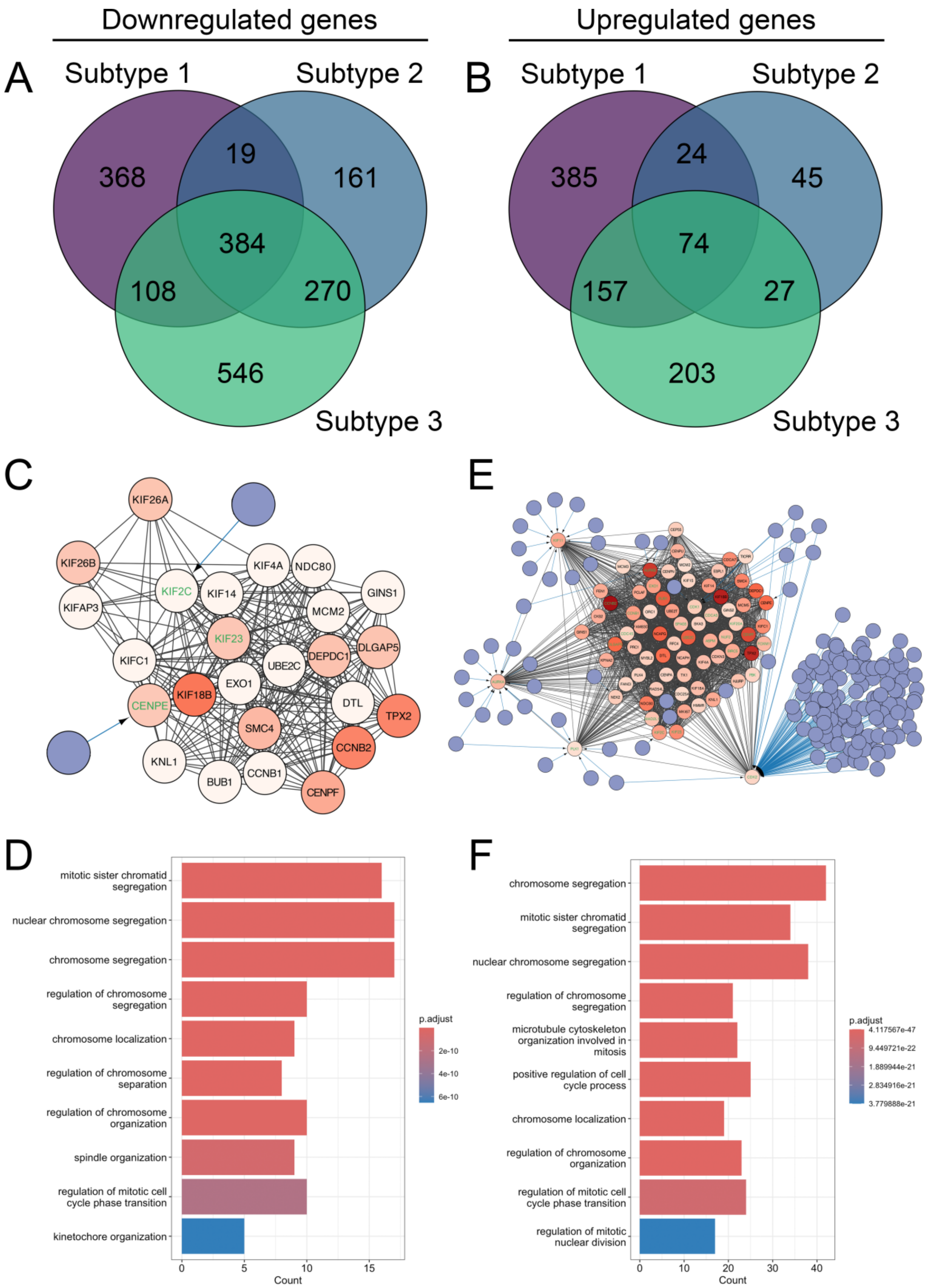

Figure S4. Differential gene expression, significant cluster identification and functional enrichment. **A**) Venn diagram of downregulated gene for each subtype. **B**) Venn diagram of upregulated genes for each subtype. **C, D)** MCODE identified, most significant cluster and the corresponding functional enrichment of biological processes in S2. **E, F**) Most significant MCODE cluster and the corresponding functional enrichment of biological processes in S3. Within the identified clusters genes have been color coded based on Log2 fold change with increased color density representing higher change values. Nodes of the identified drugs are blue and connect to predicted genes via edges. The top 10% based on degree have green labels.

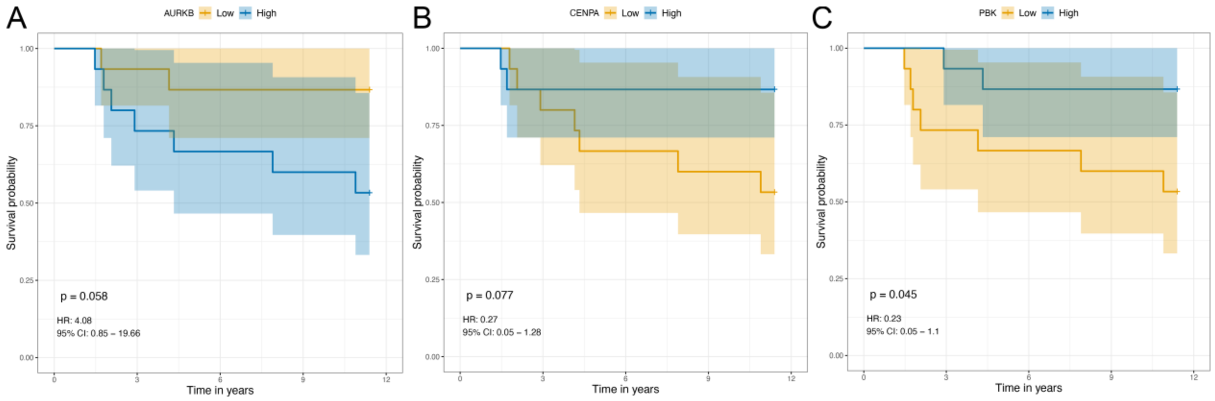

Figure S5. Survival probability and gene expression association. **A)** Survival probability of S3 patients associated with *AURKB* expression. **B)** Survival probability of S3 patients associated with *CENPA* expression. **C)** Survival probability of S3 patients associated with *PBK* expression.

	Concentration (μM)	80	120	140	160	180
**SAOS-2**	**CDI ± SD (24 h)**	0.68 ± 0.32	1.06 ± 0.04	1.28 ± 0.34	1.54 ± 0.77	1.28 ± 0.33
	**CDI ± SD (48 h)**	1.28 ± 0.07	1.34 ± 0.07	1.5 ± 0.01	1.5 ± 0.07	1.71 ± 0.07
**MG63**	**CDI ± SD (24 h)**	0.59 ± 0.32	0.4 ± 1.34	0.47 ± 0.98	0.8 ± 0.36	0.63 ± 0.13
	**CDI ± SD (48 h)**	1.08 ± 0.08	1.05 ± 0.03	1.02 ± 0.02	1.01 ± 0.04	0.99 ± 0.08

Table S1. Coefficients of drug interactions between 1 μg/ml doxoru\bicin and specified concentrations of hesperidin.

**Concentration (μM)**	160	180
**CDI ± SD**	0.55 ± 0.62	0.63 ± 0.68

Table S2. Coefficient of drug interaction (CDI) between 5-fluorouracil and specified concentrations of hesperidin on MG63 cell line after 24 h of treatment
